# Common questions and misconceptions about caffeine supplementation: what does the scientific evidence really show?

**DOI:** 10.1080/15502783.2024.2323919

**Published:** 2024-03-11

**Authors:** Jose Antonio, Daniel E. Newmire, Jeffrey R. Stout, Brandi Antonio, Maureen Gibbons, Lonnie M. Lowery, Joseph Harper, Darryn Willoughby, Cassandra Evans, Dawn Anderson, Erica Goldstein, Jose Rojas, Matías Monsalves-Álvarez, Scott C. Forbes, Jose Gomez Lopez, Tim Ziegenfuss, Blake D. Moulding, Darren Candow, Michael Sagner, Shawn M. Arent

**Affiliations:** a Nova Southeastern University, Department of Health and Human Performance, Davie, FL, USA; b Texas Woman’s University, Exercise Physiology and Biochemistry Laboratory, School of Health Promotion and Kinesiology, Denton, TX, USA; c University of Central Florida, College of Health Professions and Sciences, Orlando, FL, USA; d The Woodlands, Active Medical Solutions, TX, USA; e Nutrition, Exercise and Wellness Associates, Cuyahoga Falls, OH, USA; f Walsh University, Department of Exercise Science, North Canton, OH, USA; g School of Exercise and Sport Science, University of Mary Hardin-Baylor, Belton, TX, USA; h Indiana Tech, Exercise and Sport Performance Laboratory, Fort Wayne, IN, USA; i Stetson University, Department of Health Sciences, Deland, FL, USA; j Keiser University, Fort Lauderdale, FL, USA; k Rocky Mountain University of Health Professions, Provo, UT, USA; l Universidad de O´Higgins, Exercise Metabolism and Nutrition Laboratory. Instituto de Ciencias de la Salud, Rancagua, Chile; m Motion Human Performance Laboratory, Lo Barnechea, Chile; n Brandon University, Department of Physical Education Studies, CBrandon, MB, Canada; o The Center for Applied Health Sciences, Canfield, OH, USA; p University of Regina, Faculty of Kinesiology and Health Studies, Regina, SK, Canada; q Faculty of Medicine King’s College, Longdon, England; r University of South Carolina, Arnold School of Public Health, Columbia, SC, USA

**Keywords:** Ergogenic aid, performance, exercise, supplement

## Abstract

Caffeine is a popular ergogenic aid that has a plethora of evidence highlighting its positive effects. A Google Scholar search using the keywords “caffeine” and “exercise” yields over 200,000 results, emphasizing the extensive research on this topic. However, despite the vast amount of available data, it is intriguing that uncertainties persist regarding the effectiveness and safety of caffeine. These include but are not limited to: 1. Does caffeine dehydrate you at rest? 2. Does caffeine dehydrate you during exercise? 3. Does caffeine promote the loss of body fat? 4. Does habitual caffeine consumption influence the performance response to acute caffeine supplementation? 5. Does caffeine affect upper vs. lower body performance/strength differently? 6. Is there a relationship between caffeine and depression? 7. Can too much caffeine kill you? 8. Are there sex differences regarding caffeine’s effects? 9. Does caffeine work for everyone? 10. Does caffeine cause heart problems? 11. Does caffeine promote the loss of bone mineral? 12. Should pregnant women avoid caffeine? 13. Is caffeine addictive? 14. Does waiting 1.5–2.0 hours after waking to consume caffeine help you avoid the afternoon “crash?” To answer these questions, we performed an evidence-based scientific evaluation of the literature regarding caffeine supplementation.

## 1. Introduction

Caffeine (1,3,7-trimethylxanthine) is one of the most widely consumed substances. Caffeine is 99 percent absorbed within 45 minutes of ingestion [1]. Peak plasma concentrations occur between 15 and 120 minutes after oral ingestion. This wide variation in time may be due to variations in gastric emptying time between individuals [2]. The mean half-life of caffeine in the plasma of healthy individuals is about 5 hours. However, caffeine’s elimination half-life may range between 1.5 and 9.5 hours [3]. This wide range in the plasma mean half-life of caffeine may be due to innate individual variation and a variety of physiological and environmental characteristics that influence caffeine metabolism (e.g. pregnancy, obesity, use of oral contraceptives, smoking, altitude). Once caffeine is absorbed, there appears to be no hepatic first-pass effect (i.e. the liver does not appear to remove caffeine as it passes from the gut to the general circulation). The fact that the human body converts 70–80 percent of caffeine into paraxanthine with no apparent toxic effects following caffeine doses of 300–500 mg/day suggests that paraxanthine’s toxicological potency is low. The formation of paraxanthine and its excretion in the urine is the major pathway for caffeine metabolism and clearance [4].

Mechanistically, caffeine binds to adenosine receptors, which in turn blocks the binding of adenosine to its receptor. The blockage of adenosine receptors indirectly affects the release of neurotransmitters such as norepinephrine, dopamine, acetylcholine, serotonin, glutamate, and gamma-aminobutyric acid [5]. Another mechanism of action for caffeine involves the ability to inhibit phosphodiesterase, which prevents cAMP from being enzymatically degraded. The subsequent accumulation of cAMP then stimulates the release of hormones and neurotransmitters such as dopamine and the catecholamines (i.e. epinephrine and norepinephrine) [6]. As a result of the increase in the release of catecholamines, beta-adrenergic receptor [6] stimulation can initiate increases in adipose tissue lipolysis and subsequent fatty acid oxidation. In some acute studies, caffeine has been shown to impact lipid oxidation and metabolism [7,8].

Despite the widespread outreach of the position stand on caffeine by the International Society of Sports Nutrition [9], there continues to be persistent questions, misunderstandings, and misconceptions about caffeine. These include but are not limited to: 1. Does caffeine dehydrate you at rest? 2. Does caffeine dehydrate you during exercise? 3. Does caffeine decrease body weight and fat mass? 4. Does habitual caffeine consumption influence the performance response to acute caffeine supplementation? 5. Does caffeine affect upper vs. lower body performance/strength differently? 6. Is there a relationship between caffeine and depression? 7. Can too much caffeine kill you? 8. Are there sex differences regarding caffeine’s effects? 9. Does caffeine work for everyone? 10. Does caffeine cause heart problems? 11. Does caffeine promote the loss of bone mineral? 12. Should pregnant women avoid caffeine? 13. Is caffeine addictive? 14. Does waiting 1.5–2.0 hours after waking to consume caffeine help you avoid the afternoon “crash?”

## 2. Does caffeine dehydrate you at rest?

The general public has readily accepted that caffeine intake promotes and facilitates fluid loss through a diuresis-like effect. Is this a valid and reliable physiological response, or is this stance limited to classical research, anecdotal experiences, and hearsay? In 2003, a review paper by Maughan and Griffin proposed that a dose of caffeine of ≥300 mg (2–3 cups of coffee) would stimulate urine output in individuals who may have been “deprived of caffeine for a period of days or weeks.” [10] Furthermore, the guidelines from the United States Food and Drug Administration (FDA) recommend limiting caffeine intake to ≤400 mg/day in healthy adults, which equates to ~ 4–5 cups of coffee [11,12]. It has been reported that the average caffeine intake for Americans is ~ 165–230 mg/day, and the primary contributor to caffeine intake is in the form of coffee [13,14]. The relative dose of caffeine in more readily available drinks, such as a 12-oz can of a caffeinated soft drink, typically contains 30–40 mg, an 8-oz cup of green or black tea 30–50 mg, and an 8-oz cup of coffee closer to 80–100 mg. Caffeine found in energy drinks ranges from 40–250 mg per 8 fl. oz. [12]. However, the caffeine doses recommended by Maughan *et al*. (2003) and the FDA were not reported relative to body mass and did not take into other factors that may influence (e.g. decrease or increase) caffeine metabolism such as habituation of caffeine ingestion, pregnancy, obesity, use of oral contraceptives, smoking, altitude, and genetic polymorphism (*CYP1A2)* [15–18]. It should be noted that caffeine content can vary quite considerably from 58 to 259 mg/dose in coffee [19]. However, for this section, we will refer to the average amount (i.e. present in coffee).

Previous studies have challenged the dogma that caffeine causes dehydration [20–23]. More recently, Killer et al. (2014) gave 50 habitual male coffee drinkers (i.e. they consumed on average 3–6 cups/day or 300–600 mg/day) 200 mL of coffee four times a day at a dose of 4 mg/kg (~308 mg/trial) compared to water to observe hydration status. They observed no difference in total body water that was assessed by the gold standard protocol using deuterium oxide (D_2_O); moreover, a slight loss in body mass over the three days was observed in both the coffee and water treatments (<0.5 kg). No differences were found in voided urine volume, urine specific gravity (USG), 24-hour urinary voided volume, and urine osmolality. The only difference found in the coffee treatment was an increase in Na^2+^ excretion, which would be expected in that caffeine promotes natriuresis caused by inhibition of Na^2+^ transport along the proximal convoluted tubule. The authors suggested that Na^2+^ is known to be a determining factor of urine production; however, it is not the only factor that drives urine volume. They proposed that a portion of urine volume is also free water or solute-free water [17]. Additionally, the authors noted that no participants reported abnormal fluid losses (*i.e*. diarrhea, vomiting) in any trial. This is important in that it has been reported that caffeinated coffee induces an increase in colonic motor activity and the propagation of gastrocolic contractions compared to water. This may lead to abdominal cramping and diarrhea, which could lead to excessive fluid loss and, therefore, be a factor in hydration status [24]. A meta-analysis performed by Zhang *et al*. in 2015 assessed 16 studies that investigated the impact of caffeine-induced diuresis during resting, and exercise conditions. They found that the median caffeine consumption of these studies was ~300 mg (below FDA recommended intake), and the overall effect size (ES) for caffeine-induced diuresis was very small (0.29). However, it is evident that there is quite a high degree of variability regarding the ES. Of the 28 investigations assessed in this meta-analysis, six ESs were negative, four were trivial (<0.2), ten were small (0.20–0.49), three were moderate (0.50–0.79), and five were large (>0.80) [18]. Thus, caffeine dose was not an independent predictor of diuresis, and the claims that caffeine-induced diuresis seem unfounded [18].

Lastly, in 2017, Seal et al. found that in a sample population of 10 adult habitual coffee drinkers (1–3 cups/day) that a lower dose of caffeine (3 mg/kg or ~270 mg) compared to a higher dose (6 mg/kg or ~538 mg) ingestion in coffee compared to water showed that the higher dose of coffee intake leads to a higher voided urine volume after three hours of observation [11]. Their work corroborates with other previous investigations [25–27] showing that higher doses of caffeine (≥500 mg) seem to promote an increase in urine output. However, this higher dose of caffeine does not seem to represent what most adult Americans consume daily and may be excessive and not generalizable for a practical comparison. Moreover, Maughn and Griffin [28] state that “A profound tolerance to the diuretic and other effects of caffeine develops, however, and the actions are much diminished in individuals who regularly consume tea or coffee. Doses of caffeine equivalent to the amount normally found in standard servings of tea, coffee, and carbonated soft drinks appear to have no diuretic action.”

***In summary, previous research has suggested that a dose of ≥300 mg of caffeine may induce acute diuresis. However, recent research examining and controlling for multiple factors that may influence the validity of the study design and the responses of the participants has shown that moderate daily doses of caffeine (3 mg/kg or ~ 250–300 mg), which is above the reported average in the United States, in habitual drinkers do not seem to augment urine volume. In contrast, excessive and impractical doses of caffeine (6 mg/kg or ≥500 mg) may facilitate acute diuresis.***

## 3. Does caffeine dehydrate you during exercise?

Adenosine A_1_ receptors may contribute to the potential diuretic and natriuretic effects of caffeine via adenosine receptor A_1_ blockade in the kidneys [29–33]. However, the sporadic cases of caffeine-induced diuresis should not be confused with 24-hour fluid balance [34]. Killer et al.. (2014) [17] reported no difference between coffee and water for 24-hour urine volume, void volume, urine specific gravity (USG), or osmolality when moderate coffee drinkers (300–600 mg/day) consumed coffee vs. water [17]. Similarly, Armstrong [34] found no differences between low (3 mg/kg) and moderate (6 mg/kg) caffeine intake for 24-hour urine volume, color, USG, osmolality, or sodium [34]. These outcomes [17,29,34,35] indicate caffeinated beverages do not negatively impact fluid retention following exercise. The potential diuretic effect of caffeine can be moderated by fluid balance, genetics, and exercise [18,29,35]. Ganio et al.. (2011) [36] found caffeine (6 mg/kg) enhanced 105 min of cycling performance compared to placebo in both cool and hot conditions when water intake controlled changes in body mass [36]. There was no difference in USG, urine, or serum osmolality between treatments, demonstrating the independent effects of temperature on caffeine performance when hydration is managed [36].

Several studies have examined caffeine intake (5.3–9 mg/kg) during endurance exercise in the heat [36–40] in recreational and endurance-trained, non-heat acclimated males [36–39]. Millard-Stafford et al.. (2007) reported no significant differences in fluid retention or urine output between water, carbohydrate-electrolyte solution (CES), or CES with caffeine during 2 hours of cycling and 15-minute max effort in the heat [39]. Rectal temperature increased 0.19–0.29° C with caffeine, corresponding to higher relative exercise intensity [39]. Beaumont and James (2017) found caffeine increased work production during 30-min maximal effort after 60 min of moderate cycling in the heat compared to placebo [37]. However, the estimated sweat rate and body mass loss were greater with CAFF [37]. While most found no significant difference between caffeine and placebo for fluid retention, urine output, percent dehydration, or core temperature when appropriate fluids were consumed, marginal ~ 0.2–0.3° C increases in body temperature occurred with caffeine [36–40].

Interindividual variability in response to caffeine is often high due to genetics and biological sex [35,41]. Overall, there appears to be attenuated clearance in females vs. males, resulting in higher caffeine and lower paraxanthine levels in females [41]. However, McLean and Graham (2002) [41] found no sex difference for caffeine pharmacokinetics or exercise-induced dehydration [41]. In their study, 6 mg/kg of caffeine resulted in no differences in caffeine metabolism in females in the follicular and luteal phases at rest and during exercise [41]. Plasma caffeine was higher and paraxanthine lower in females vs. males over eight hrs., yet no sex difference occurred for pharmacokinetics or dehydration during exercise [41]. The CYP450 enzyme system may saturate earlier in women but not impact caffeine metabolism during exercise [41].

Fiala et al. (2004) [42] reported no difference in hydration status when a mixed group of women and men rehydrated with caffeinated vs. caffeinated-free cola between exercise sessions over three days [42]. Urine color increased slightly with caffeine post-exercise, but other markers like plasma volume, sweat rate, plasma/urine osmolality, and urine volume/USG did not differ between treatments [42].

***In summary, sweat rate, hydration strategy, and genetics appear more influential on hydration status than reasonable caffeine intake alone. The diuretic effect may be minor or non-existent; however, appropriate fluid intake prevents negative effects on fluid balance. A potential confounder is whether caffeine is consumed as a pill or as part of a multi-ingredient pre-workout supplement with other ingredients and extra fluid.***

## 4. Does caffeine decrease body weight and fat mass?

Several studies have investigated the potential association between caffeine and fat oxidation and metabolism. Notably, many studies involved an exercise trial, which may impact fat oxidation differently than when caffeine is given in an inactive state. A study provided active, non-overweight, caffeine-naive men with anhydrous caffeine from green coffee beans at a dose of 3 mg/kg (210 mg in a 70 kg man) prior to a graded exercise test on a cycle ergometer at four different intervals, two in the morning and two in the evening. Regardless of the time of day, caffeine was shown to significantly increase the estimated value for fat oxidation [43]. Another study involving one hour of self-paced cycling in active and non-overweight men and women after ingesting 3 mg/kg of caffeine [44] observed a significant increase in the rate of energy expenditure but did not increase the rate of fat oxidation.

A systematic review and meta-analysis was recently conducted to better understand the effects of acute caffeine ingestion (ranging from 2 to 7 mg/kg of body mass) on fat oxidation during exercise [45]. Results showed that caffeine significantly increased the rate of fat oxidation and reduced the respiratory exchange ratio. Further, a dose-response effect of caffeine on the rate of fat oxidation was evident, with ≥3 mg/kg of caffeine significantly increasing the rate of fat oxidation during exercise. Additionally, the ability of caffeine to enhance fat oxidation during exercise was higher in sedentary or untrained individuals than in trained and recreational athletes.

Several studies have been conducted investigating the longer-term effects of caffeine on body weight loss due to reductions in fat mass. Many of these studies showed favorable results but used: 1) caffeine doses considerably higher than many other studies not finding favorable results or 2) other thermogenic-related compounds [i.e. ephedra, epigallocatechin gallate (green tea), citrus aurantium (bitter orange), etc.] in conjunction with caffeine that most likely synergized the effect of caffeine, which make interpreting the results for caffeine as a single ingredient difficult.

Two randomized control trials totaling 87 inactive, overweight participants ingesting green coffee bean extract at a range of 400 mg/day [46] and 1,000 mg/day [47] for eight weeks found no significant difference in body weight when compared to a placebo. However, a four-week study involving overweight participants ingesting approximately 700 mg/day of freshly brewed Arabica coffee demonstrated significant decreases in body weight [48]. Another study [49], 12 weeks in duration with overweight participants consuming coffee with a caffeine content of approximately 500 mg/day, found significant decreases in body weight and body fat percentage. Moreover, a meta-analysis conducted by Tabrizi et al. [50] concluded that “caffeine intake might promote weight, BMI and body fat reduction.”

***In summary, in longer-term caffeine studies examining weight loss, conflicting results may be due to several limiting issues, such as the standardization of dietary intake. Another variable is whether the participants were caffeine naïve prior to the study. If not, perhaps they are habituated to the caffeine dose during the course of the study, thereby minimizing weight loss. The other issue is the dose of caffeine utilized and perhaps whether overweight or non-overweight participants were involved.***

## 5. Does caffeine affect upper vs. lower body performance/strength differently?

Caffeine’s effects on performance, especially between upper and lower body strength, have garnered much interest. The impact seems to depend on several factors, such as dosage, muscle group size, and type of activity.

Some studies show that caffeine improves lower body performance. For example, Ruiz-Fernández et al. found that caffeine increased velocity, power, and force development more in back squats than in the bench press, particularly at higher weights [51]. Similarly, Tallis and Yavuz reported an increase in the knee extensor, but not the elbow flexor force, after caffeine [52]. Duncan et al. [53] showed that both upper and lower body anaerobic performance was enhanced 60 minutes following caffeine consumption (5 mg/kg).

However, other research highlights marked upper body benefits. Andrea et al. found improved peak and mean upper body power from caffeine without similar lower body effects [54]. Beck et al. [55] and Goldstein et al. [56], respectively, noted enhanced maximal upper body strength in trained men and women after caffeine. Furthermore, Sabol et al. reported that a dose of caffeine of 6 mg/kg was optimal for upper-body ballistic performance, suggesting that dosages may differ by muscle group [57].

Interestingly, some studies suggest that caffeine’s effects depend less on the upper or lower body and more on dose and individual variation. Degrange et al. found that speed and power output increased in upper and lower body exercises after caffeine intake [58]. Timmins and Saunders reported an improvement in maximum torque in all muscles with slightly greater effects in larger muscle groups [59]. Lane and Byrd noted that caffeine maintained upper body power but not in the lower body [60].

A few meta-analyses have produced different conclusions regarding the effects of caffeine on upper and lower body strength. Warren et al. [61] initially suggested that caffeine has a significantly greater impact on lower-body muscle groups, resulting in a fourfold to sixfold increase in strength compared to upper-body muscle groups. However, a subsequent analysis by Grgic et al. [62] contradicted this, proposing that caffeine intake leads to a noticeable increase in upper body strength but no corresponding effect on lower body strength. Building on this research, Ferreira et al. [63] conducted a more recent meta-analysis, which found that caffeine consumption leads to an increase in maximal strength for upper body exercises, particularly the bench press, by approximately 2.01 kilograms. However, they found no significant impact on maximal strength in lower body exercises, such as the leg press. These conflicting results highlight the complexity of caffeine’s ergogenic effects and emphasize the need for further comprehensive research. Future investigations should aim to elucidate the underlying mechanisms that contribute to the varied responses in muscle group strength to caffeine, ultimately providing a more nuanced understanding of its role in exercise and athletic performance.

The dose-response relationship further complicates matters. Although Rocha et al. [64] found no benefits with 5 mg/kg caffeine, Andrea et al. saw upper body improvements at 7 mg/kg [54]. Ferreira et al. suggested that 8 mg/kg is optimal for trained individuals, which may be too high of a dose for most people [65]. Thus, ideal doses probably differ depending on the person and activity.

***In summary, caffeine’s ergogenic effects on upper versus lower body strength depend on dose, individual differences, muscle group size, and activity type rather than a clear bias. The advantages are situational and unique rather than inherent in either the upper or lower body.***

## 6. Is there a relationship between caffeine and depression?

While many people turn to caffeine to enhance alertness and combat fatigue, its impact on mental health, particularly its relationship with depression, has been a subject of extensive research and debate [66]. The relationship between caffeine and depression is complex and multifaceted, with both positive and negative effects depending on various factors [66–75]. Caffeine has been linked to the release of certain other neurotransmitters, including dopamine and serotonin, which are associated with mood regulation and feelings of well-being [71]. This has led researchers to examine the relationship between mood and caffeine intake [74]. Some studies have even found an inverse relationship between coffee consumption and depression risk [76], implying that individuals who regularly consume moderate amounts of caffeine are less likely to develop depressive symptoms [77]. Moderate caffeine is defined as up to 400 mg/day [73].

However, the relationship between caffeine and depression is not entirely positive [72]. Excessive caffeine intake can lead to several negative consequences for mental health. For instance, it can exacerbate symptoms of anxiety, which often co-occur with depression [70,72,75,78–80]. High doses of caffeine [81] can trigger restlessness, nervousness, and even panic attacks in susceptible individuals, making them more vulnerable to rebound and depressive episodes [82,83]. It should be noted with regard to panic attacks that 61% of patients with panic disorders suffered panic attacks after consuming 480 mg of caffeine; on the other hand, control subjects did not experience a panic attack [83].

Moreover, excessive caffeine consumption can disrupt sleep patterns. A review by Barnard et al. posited that “evening (≥5 p.m.) caffeine intakes >2 mg·kg − 1 body mass decreased sleep duration and sleep efficiency, and increased sleep latency and wake after sleep onset [84]” Poor sleep quality is a well-established risk factor for depression and can contribute to the development and persistence of depressive disorders [85]. The effects of caffeine on depression also vary depending on individual sensitivity and tolerance. Some people are more sensitive to caffeine’s stimulating effects and may experience jitteriness and increased anxiety even with low to moderate consumption [86]. On the other hand, individuals who are more susceptible to or have built up a high tolerance to caffeine may require larger doses to achieve the same stimulating effects, potentially leading to a cycle of increased consumption and heightened sensitivity to caffeine’s negative effects [79]. Furthermore, caffeine withdrawal can be another issue to consider in the context of depression. When regular caffeine consumers suddenly reduce their intake or quit caffeine altogether, they can experience withdrawal symptoms such as headaches, irritability, and fatigue, worsening depressive symptoms [87].

***In summary, the relationship between caffeine and depression is highly individualized*** [86***]. While moderate caffeine consumption may provide temporary relief from some depressive symptoms and even have potential mood-enhancing effects for some individuals, excessive or poorly managed*** [88] ***caffeine intake can exacerbate anxiety*** [66***], disrupt sleep*** [85***], and lead to negative mental health outcomes. It is crucial for individuals with depression*** [89] ***to be mindful of their caffeine consumption and its impact on their well-being, as well as to seek professional guidance if necessary, as well as consider any other physical diagnoses present*** [74].

## 7. Can too much caffeine kill you?

Due to its broad presence in the food supply, the Food and Drug Administration (FDA) has recommended a daily limit of 400 mg or 0.4 grams [90]. Further, “the FDA estimates toxic effects, like seizures, can be observed with rapid consumption of around 1,200 milligrams of caffeine, or 0.15 tablespoons of pure caffeine” [90]. Observing extremes (i.e. death), the median lethal dose (LD50) of caffeine is informative but not actionable when deciding how dangerous caffeine intake may be. LD50 is a statistically derived amount of a substance that can be expected to cause death in 50% of the animals when given by a specified route as a single dose and observed for a specific period of time. A review of related studies on rodents stated that “the most accurate estimate of the acute LD50 of caffeine administered orally in male albino rats is hereby reported to be 367 mg/kg” [91]. Of course, this lethal value is absurdly high from a food or dietary supplement perspective, making the FDA’s determinations more applicable for consumers, for which there is indeed some risk. The FDA noted that “Products consisting of or containing only pure or highly concentrated caffeine have been linked to at least two deaths in the United States in the last few years and continue to present a significant public health threat” [92]. In addition to such governmental and scientific concerns over toxicity with high doses of caffeine, reports of caffeine-related deaths have made the news. A news article reported a death that they described as a “miscalculation that led to a massive overdose” linked to high doses of caffeine powder [93]. According to Cappelletti et al., [93], “Five caffeine-related deaths (5%) among athletes have been described in the literature; these subjects were two amateur bodybuilders, a basketball player, and a wrestler. The age ranged from 18 to 44. In all cases, the cause of death was attributed to cardiac arrest due to ventricular fibrillation” [93]. Similarly, in a review and case study by Willson [94], the author stated that ventricular fibrillation is most often the cause of death but that with regard to myocardial infarction, coronary artery vasospasm has also been proposed as a cause of death. Kerrigan and Lindsey reported that a fatal dose of caffeine typically exceeds five grams [95]. In a case report by Jabbar et al., a 39-year-old male died of an overdose of approximately 22 grams of caffeine [96].

Given the vast numbers of people consuming caffeinated products in their many forms, and the typical doses present in them, it is possible to see that although potentially lethal upon overdose (e.g. via miscalculated intake of concentrated liquids or powders), caffeine present in dietary products is not particularly deadly. Indeed, scientists at the FDA, although concerned about pure or highly concentrated caffeine, have stated, “caffeine can be part of a healthy diet for most people, but too much caffeine may pose a danger to your health [90].

***In summary, caffeine can be lethal when overdosed, but this is not typically possible in healthy persons when consumed in caffeinated beverages like coffee and tea.*** ***At a potentially lethal dose of >5000 mg or 5 grams, it is unlikely that an individual would overdose via the consumption of caffeinated beverages.***

## 8. Are there sex differences regarding caffeine’s effects?

Biological males and females have clear anatomical, physiological, and psychological differences. These differences lead to separate nutritional recommendations for males and females. The Dietary Guidelines for Americans provide separate recommendations for substances such as fiber, iron, and alcohol [97]. Sex differences in response to pharmaceutical drugs are well elucidated. Females are more likely to suffer from adverse events or overdose in response to medications. [98] This is due to increases in drug concentration and rates of drug clearance [98]. Caffeine is a naturally occurring stimulant that is classified as a psychoactive drug [1,9,92,99]. It exerts its effects on the central nervous system via the adenosine receptors. Due to the sex differences in pharmacokinetics and pharmacodynamics, some believe differences in caffeine metabolism are present.

Females have lower activity of CYP1A2, the isoenzyme responsible for caffeine metabolism in the liver [98,100]. Lower CYP1A2 activity interferes with caffeine metabolism [35,100]. Despite this difference, research suggests caffeine metabolism is the same in males and females. McLean et al. [41] reported a “trend for higher plasma caffeine concentration” in females; however, no sex difference in area under the curve (AUC), peak, or time to peak caffeine concentration was observed. Following the consumption of caffeine (3 mg/kg), Skinner et al. [92] reported similar plasma concentrations between males and females. Due to its stimulant properties, caffeine increases heart rate in most individuals. However, Hartley et al. [101] reported no difference in heart rate and blood pressure, and Kurokawa et al. [102] reported no differences in heart rate between males and females after ingesting caffeine; however, a greater cardiac output was observed in females. Conversely, Temple et al. [103] reported higher diastolic blood pressure and elevated heart rate in females after caffeine consumption despite administering a lower dose of caffeine (2 mg/kg). Clark et al. [104] conducted a study on the effect of a caffeine-containing (140 mg) energy drink on heart rate variability (HRV) during graded exercise tests and short-term rest. No sex differences in HRV were reported.

The literature on sex differences in response to caffeine consumption is somewhat mixed. Adan et al. [105] reported sex differences in response to caffeinated and decaffeinated beverages. Low-dose caffeine intake (100 mg) had a greater effect on subjective ratings of sleepiness and effects of caffeine in males. Interestingly, females reported greater effects following the consumption of decaffeinated beverage [105]. Domaszewski et al. [106] reported an increased occurrence of self-reported positive effects of caffeine in males compared to females. Positive and negative effects were assessed via the “Negative and Positive Effects after Caffeine Ingestion Questionnaire (QUEST).” A study evaluating cognitive performance via mental calculations reported that both sexes improved performance with no significant differences between females and males [102]. A study examining the ergogenic effects of caffeine on time to exhaustion reported both males and females improved following the ingestion of caffeine (3 mg/kg) [92]. Similarly, Sabblah et al. [107] found no sex differences in maximal strength and muscular endurance. Hou-Yu Chen et al. [108] reported caffeine ingestion resulted in a greater decrease in delayed onset muscle soreness (DOMS) in males compared to females. DOMS negatively impacts performance; thus, males may indirectly experience a greater ergogenic effect of caffeine. Anderson et al. [109] conducted a study using only female subjects. Competitive oarsman significantly improved performance after ingesting 6 or 9 mg/kg. These studies suggest caffeine has ergogenic effects for both males and females. According to a systematic review by Jimenez et al., “the magnitude of the ergogenic benefits obtained with caffeinated drinks seems similar in female and male athletes [110].”

***In summary, the literature regarding sex differences in response to caffeine is mixed. Possible contributing factors such as estradiol***^***103***^ ***and oral contraceptive use decrease*** [111] ***CYP1A2 activity, which is directly involved in caffeine metabolism. Menstruation and subsequent variations in hormones and other physiological differences may account for the reported sex differences. Likewise, caffeine’s effects on sports performance are mixed. Caffeine is an ergogenic aid, and even the placebo effect of caffeine improves sports performance irrespective of sex, with some studies reporting greater performance advantages in males*** [102,106–108].

## 9. Does habitual caffeine consumption influence the performance response to acute caffeine supplementation?

The common belief that habitual caffeine intake influences the performance response to acute caffeine supplementation is often attributed to an upregulation of adenosine receptors, which may attenuate the effect of caffeine [112]. Although this study by Fredholm is often cited, the increase in the number of adenosine receptors was not demonstrated in humans but in rats after consuming 20 mg/kg/day for seven days. It has been theorized that over time, a higher dose of caffeine would be required to elicit the same effects as previously experienced. Soon after this publication, Van Soeren and Graham [113] explored the potential impact of habitual caffeine intake on performance responses to acute caffeine supplementation. They tested caffeine-consuming (761.3 ± 11.8 mg/day), recreational athletes (*n* = 6) following short-term withdrawal (0 days, two days, and four days) from caffeine and found a 6 mg/kg dose of caffeine to result in no significant differences in hormonal (epinephrine, norepinephrine, insulin), metabolic (free fatty acids, glycerol, glucose, lactate), or performance (cycle to exhaustion at 85% VO_2_max) measures between any of the withdrawal periods.

More recent evidence demonstrates no effect of habitual caffeine consumption on the ergogenic response to caffeine [113–123]with fewer studies having a lesser effect of acute caffeine intake on performance for habitual caffeine consumers [124–126]. The lesser benefits seen in some studies may be related to the dose of caffeine provided during the performance trials being similar to their habitual intake level of 3 mg/kg/d. Pickering and Grgic [127] stated that a 6 mg/kg dose or higher of caffeine may be required for habitual caffeine consumers. According to Zhang et al. [128] a lower dose (≤3 mg/kg) of caffeine is sufficient to impact the CNS; however, a high dose of 6–9 mg/kg may be necessary for peripheral effects on performance.

Another reason for the lack of consistency with results may be related to the levels of caffeine used to classify individuals as low and high users of caffeine. The ranges vary greatly from low users at 20 mg/d [117] to 165 mg/day [120] and high users at ≥100 mg/day [122] to 415 mg/day [116]. The lack of consistency in habitual caffeine intake adds to the uncertainty regarding the impact of regular caffeine consumption on the ergogenic effects of caffeine.

A recent meta-analysis to determine whether habitual caffeine consumption alters the ergogenic effect of caffeine was conducted by Carvalho et al. [115]. Of the 246 studies that met the initial inclusion criteria, only 60 studies reported habitual caffeine consumption of the subjects. The meta-analysis concluded that habitual caffeine consumption had no influence on the ergogenic effect of caffeine. However, with 76% of the studies not including information about habitual caffeine intake, there is a large gap in what is reported and, thus, what is known about the impact of habitual caffeine intake on exercise performance. In an elegant review by Tallis et al., [129] the authors suggest that “caffeine is ergogenic irrespective of the typical caffeine consumption habits.”

***In summary, the preponderance of the evidence suggests that habitual caffeine intake does not negatively impact performance following acute caffeine administration. It may be that a dosage of 6–9 mg/kg of caffeine is necessary to affect performance. However, with the majority of studies not reporting habitual caffeine intake, more research is needed.***

## 10. Does caffeine work for everyone?

Caffeine supplementation use is widespread among exercising individuals [9]. However, whether caffeine works for everyone to enhance exercise performance is debatable [9,130–132]. Caffeine is one of the few well-established ergogenic aids [9,133] that consistently enhances various aspects of exercise performance, including muscular endurance, muscular power, muscular strength, sprinting, jumping, throwing, and endurance [9,133,134]. The overall magnitude of the effect of caffeine appears to be associated with the exercise task [9,133]. Furthermore, within a specific exercise task, there are large inter-individual differences [35]. Several reviews have examined potential moderating effects, including genetic polymorphisms (CYP1A2 and ADORA2A genotypes), training status, dose, time of day, and sex [9,135,136].

Caffeine is rapidly metabolized in the liver via the cytochrome p450 enzyme [137]. The cytochrome p450 family 1 subfamily 2 (CYP1A2) gene is responsible for > 90% of caffeine metabolism [137]. As such, genetic variations in this gene are purported to influence the rate of caffeine breakdown. Individuals with homozygous A/A alleles are identified as fast caffeine metabolizers, whereas C allele carriers (A/C and C/C) are known as slow metabolizers [9]. Therefore, this CYP1A2 polymorphism may influence the ergogenic effects of caffeine on an individual. Currently, results are mixed, with some showing that fast metabolizers outperform slow metabolizers [138–140], while others found no effect [141,142] and one study reported that slow metabolizers outperformed fast metabolizers [143]. Overall, there is no consensus that this particular gene determines the responsiveness of caffeine supplementation [9,135]. Another gene, ADORA2A, which encodes the adenosine receptor A2A, has been purported to influence the responsiveness to caffeine [9]. Currently, limited research has explored the ergogenic effects of various polymorphisms in the ADORA2A gene. Dos Santos and colleagues [144] found that neither the ADORA2A nor CYP1A2 genotypes influence the acute caffeine effects on performance in 90 adolescents. Male adolescents independent of their genotype C>T ADOA2A (TT homozygous or C_ADORA2A_ allele) or C< T (AA homozygous or CCYP1A2 allele carriers) enhanced handgrip strength, countermovement jump (CMJ), spike jump, sit-ups and the distance covered on the Yoyo IR1 test after consuming 6 mg/kg caffeine when compared with placebo [144]. These results suggest that the ergogenic effects of caffeine are not influenced to a large extent by genetic factors.

Southward et al. [131] investigated responders’ and non-responders’ reactions to caffeine. They reported that up to 33% of individuals did not respond to caffeine (that is, they performed worse on a single exercise task following caffeine ingestion compared to a placebo and were classified as “non-responders”) [131]. This appeared to be an overestimation due to various methodological considerations, including the reliability of the exercise protocol, the lack of multiple exercise tests, the dosing strategy, and the repeatability of the effect of caffeine [130,132]. Grgic [130] performed a correction based on the coefficient of variation (used as an indicator of test re-test reliability) for the performance tasks and noted that the initially reported value of 33% of individuals being classified as non-responders was subsequently reduced to only 5%. Further, this does not indicate that the 5% are true “non-responders” to caffeine; it may be the case that these individuals may require a higher dose or a different supplement timing protocol to achieve an ergogenic effect. Del Coso and colleagues [132] demonstrated that all individuals had some increase in performance (although highly variable), suggesting that caffeine enhanced performance (at least to some degree) in everyone. Certainly, a pressing limitation regarding investigations in this area is that the “performance enhancing effects of caffeine may not be repeatable between days,” and thus, despite caffeine being an ergogenic aid, one is not likely to observe an ergogenic effect in all circumstances [145].

***In summary, there is clear evidence that a large inter-individual variability exists with regard to the ergogenic response to caffeine ingestion. Furthermore, the performance-enhancing effects of caffeine are not always repeatable. Presently, if non-responders to caffeine do exist, it is likely rare. Future research using repeated tests with multiple tasks and dosing strategies is required to confirm the responsiveness of an individual.***

## 11. Does caffeine cause heart problems?

The scientific literature documents several potential cardiovascular benefits of caffeine. Acute ingestion of caffeine has been consistently demonstrated to improve cognitive performance, particularly in the realms of attention and vigilance [146]. Improved cognitive function can indirectly contribute to cardiovascular well-being by promoting healthier lifestyle choices, such as increased spontaneous physical activity [147]. Regarding direct vascular effects, some research suggests that caffeine, in moderate doses, can lead to short-term improvements in endothelial function by promoting nitric oxide production [148]. This is critical because endothelial dysfunction is a well-established precursor to atherosclerosis, one of the primary underlying causes of various CVD. Moreover, epidemiological studies indicate long-term caffeine consumption might protect against specific cardiovascular conditions. For instance, habitual caffeine use via coffee has been inversely related to the incidence of stroke and heart failure [149]. Furthermore, caffeine intake from coffee has been shown to be associated with a lower risk of developing Type 2 diabetes, a major cardiovascular risk factor [150].

Despite these promising effects, the potential cardiovascular risks linked to caffeine should not be disregarded. For starters, acute ingestion of caffeine is often accompanied by a transient increase in heart rate and systolic and diastolic blood pressure, a phenomenon confirmed across multiple research studies [151]. Also, caffeine can elevate myocardial oxygen demand, which, under certain conditions, could act as a trigger for myocardial ischemia or exacerbate preexisting cardiovascular conditions [152].

Some longitudinal studies suggest an association between excessive caffeine consumption and adverse cardiovascular outcomes. High caffeine intake has also been correlated with increased arterial stiffness, a known risk factor for CVD [153]. A heightened risk of atrial fibrillation, the most common form of cardiac arrhythmia, has also been reported among heavy caffeine users [154].

Clear interpretation of the current body of evidence is complicated by several factors. The first major issue is the extensive variability across research designs, which include cross-sectional studies, randomized controlled trials, and prospective cohort studies, each with its own limitations [155,156]. The dosage of caffeine administered and the population subsets studied also introduce variability and, sometimes, conflicting outcomes [155]. The field is further complicated by genetic variations in the metabolism of caffeine, primarily modulated by polymorphisms in the CYP1A2 gene [157]. Some individuals are “fast metabolizers” who are less susceptible to the adverse cardiovascular effects of caffeine, while “slow metabolizers” may be at increased risk [157].

Current guidelines, such as those issued by the FDA, generally consider a daily caffeine intake of up to 400 mg as safe for most adults [158]. Nevertheless, the need for personalized risk assessment cannot be overstated, especially among populations with preexisting cardiovascular conditions or heightened susceptibility to other CNS stimulants and/or preexisting (but otherwise unknown) CVD [159]. Given the intricacies of caffeine’s impact on cardiovascular health, more rigorous, large-scale longitudinal studies are essential for clarifying all potential cardiovascular risks. These studies should aim to minimize confounding factors and focus on different metabolic phenotypes to provide more specific recommendations [160].

***In summary, the overall impact of caffeine on an individual’s cardiovascular risk profile is likely to be influenced by a myriad of factors, including dosage, duration of consumption, mode of consumption (e.g., coffee, tea, etc.), and individual metabolic and genetic differences. In addition, much of one’s caffeine consumption is via coffee. It is known that regular coffee consumption confers a myriad of benefits. The current FDA guidelines suggest consuming no more than 400 mg daily. Clearly, the effects of 400 mg will differ if one’s body mass is 60 kg vs. 100 kg. Moreover, this must be tempered with the fact that many exercising individuals may exceed that dose when caffeine is used as an ergogenic aid.***

## 12. Does caffeine promote the loss of bone mineral content?

Osteoporosis is a common endocrine and metabolic disease of the skeletal system characterized by a decrease in bone mineral density (BMD), which increases fracture risk [161]. Osteoporosis and fractures resulting from weakened bones pose significant global health challenges, impacting millions of individuals [162]. With the aging population, the prevalence of osteoporosis and fractures is likely to rise [163]. To safeguard long-term bone health, one important assessment marker is BMD [161]. Commonly recognized factors negatively influencing BMD include insufficient physical activity, low body mass index, smoking, alcohol consumption, inadequate intake of calcium, and vitamin D deficiency [161,164–166]. However, the relationship between dietary caffeine, BMD, and fracture risk is unclear.

Research regarding the safety and efficacy of caffeine on measures of bone and fracture risk is mixed. A recent meta-analysis by Zeng et al. [167] involving > 7000 participants with osteoporosis and > 390,000 participants at risk for fracture (age range 19–93 years) showed that high coffee consumption was associated with a lower risk of osteoporosis, independent of bone assessment location (pooled odds ratio: 0.79 [95% CI: 0.65 to 0.92]). The overall consumption of coffee and hip fracture incidence was non-significant. However, the quantity of coffee did appear to influence hip fracture risk, independent of sex (*p* = 0.004). Compared to no coffee consumption, the relative risk (95% CI) of hip fracture in those who consumed 0.5, 2.5 cups, 5 cups, and > 9 cups of coffee per day was 0.96 (0.92 to 0.99), 0.89 (0.83 to 0.95), 0.93 (0.86 to 1.01) and 1.10 (0.76 to 1.59) respectively. Thus, consuming < 4 cups of coffee daily was associated with a lower risk of hip fracture compared to ≥ 9 cups per day. A limitation of this meta-analysis was that the absolute dosage of caffeine contained in the coffee was not provided. However, a previous meta-analysis involving > 30000 females (age range: 40 to 76 years) showed that 330 mg of caffeine per day (or approximately 4 cups of coffee) was associated with an increased risk of fracture compared to females consuming less than 2 cups of coffee per day (or <220 mg of caffeine) [168]. The authors also showed that 4 cups of coffee daily was associated with reduced BMD in > 60000 females (~54 years) [168].

Mechanistically, these potential dose-dependent adverse effects from caffeine (and/or coffee) on bone mineral and fracture risk (especially in older adults) may be related to its effect on adenosine and vitamin D receptors, osteoblast activity, and calcium homeostasis. There is some evidence that caffeine negatively affects BMD through nonspecific antagonism of adenosine receptors [169]. Caffeine inhibits all four types of adenosine receptors (A_1_, A_2A_, A_2B_, and A_3_), which are expressed in both undifferentiated osteoblast precursors and differentiated osteoblasts, cells involved in bone formation [170]. Furthermore, caffeine has been shown to suppress vitamin D function and alter the expression of the vitamin D receptor (VDR), which may adversely affect BMD [171]. Specifically, caffeine reduces the 1,25(OH)D-induced VDR expression in human osteoblasts, as well as 1,25(OH)D-induced alkaline phosphatase activity, a marker of osteoblast activity [171]. In addition to changes in the bone remodeling process, alterations in calcium metabolism may also adversely influence BMD [171–173]. Using a rodent model of osteoporosis, Chen and Whitford [174] demonstrated that caffeine consumption increased urinary excretion of calcium, which was associated with decreased bone mineral content over time. However, this effect may be dose-dependent.

In contrast, some research shows positive associations between caffeine and/or coffee ingestion on measures of bone mineral. For example, consuming coffee (>5 times per week) was associated with increased t-scores in pre-menopausal women and older males. The authors speculated that these bone benefits may be related to the high antioxidant and anti-inflammatory properties of coffee [175]. Furthermore, coffee contains tannic acid, which has been shown to promote bone health in animal studies [176]. Others have found no association between caffeine and/or coffee consumption and change in bone mineral density or fracture risk [169,177,178].

***In summary, there is evidence to suggest that ≤ 4 cups of coffee (≤400 mg of caffeine) is the threshold for concern regarding BMD and/or fracture risk, primarily in females. It is unclear whether any presumed effect is from beverages such as tea, coffee, or energy drinks compared to caffeine alone. Moreover, the lack of RCTs on this issue makes it difficult to arrive at a definitive conclusion. Additional clinical research, specifically RCTs, is needed to explore the potential dose-response relationship between caffeine consumption and bone health and investigate relevant confounding variables (i.e. diet, population, and mode of caffeine delivery [e.g. coffee, energy drinks, tea, soft drinks, etc.) before drawing any firm conclusions.***

## 13. Should pregnant women avoid caffeine?

Pregnant women commonly consume caffeine to avoid fatigue or as part of a habitual routine. About 80% of pregnant women consume caffeine orally on a daily basis [179]. Epidemiological studies have revealed that caffeine consumption during pregnancy is associated with adverse gestational outcomes, yet the underlying mechanisms remain obscure. Caffeine is lipophilic enough to freely transfer across all biological membranes, including the blood – placental barrier, while neither the fetus nor the placenta has the enzymes for its metabolism. Caffeine absorbed by mothers may also accumulate in oviductal or uterine fluid environments, which potentially affects embryonic development and generates adult-onset diseases [180].

Epidemiological studies showed that caffeine consumption during pregnancy was associated with intrauterine growth retardation (IUGR)/low birth weight [181], subfertility, and spontaneous abortion. A ′safe′ maximum caffeine dosage for gestational health has been previously claimed: daily intake of less than 300 mg of caffeine (approximately three cups of coffee) during pregnancy was deemed unlikely to harm gestational health. However, this ′safe′ dosage is being reevaluated based on increasing evidence, which has shown that even daily doses of less than 300 mg may increase the risk of pregnancy failure. Furthermore, other studies found that even a daily intake as low as 100–200 mg during pregnancy is associated with an increased risk of miscarriage, fetal growth restriction, low birth weight, as well as increased risks to the offspring, including cognitive development impairments, overweight, and obesity [182]. These studies raise concern that there may be no absolute safe threshold of caffeine consumption during pregnancy. In addition, recent studies have begun to provide evidence showing that caffeine exposure during pregnancy can cause adverse effects on the offspring or even subsequent generations [183], suggesting possible epigenetic regulation through early embryonic or fetal germ cells via the maternal environment.

Notably, caffeine’s effects on pregnancy outcomes have been shown to be highly variable between individuals, with the individual variation in caffeine response involving the sensitivity of adenosine receptors and regulation of CYP1A2, the rate-limiting enzyme in caffeine metabolism [184].

A review of the available literature shows that the substantial majority of findings from observational studies and meta-analyses is that maternal caffeine consumption is reliably associated with major negative pregnancy outcomes. The maximum safe dose of caffeine in their diet is 200 mg per day, according to the NIH and European Food Safety Authority. The World Health Organization and European Food Safety Authority recommend that daily caffeine consumption remain below 200–300 mg as a safe dosage for pregnant health. Current science suggests that caffeine consumption in human pregnancies greater than the recommended doses should be avoided [183].

***In summary, the consensus derived from observational studies and meta-analyses is that maternal caffeine intake consistently links to adverse pregnancy outcomes*** [185***]. The cumulative scientific evidence suggests that pregnant women and those considering pregnancy should be advised to abstain from caffeine.***

## 14. Is caffeine addictive?

There seems to be a common assumption that caffeine is an addictive substance. However, this is a somewhat complex issue due to semantic considerations around the concept of addiction as well as a lack of clarity on the reward mechanisms that would need to be involved. According to the Diagnostic and Statistical Manual of Mental Disorders, Fifth Edition [186], caffeine withdrawal is an officially recognized diagnosis, as are caffeine-induced disorders and caffeine intoxication. The DSM-V also introduced criteria for caffeine use disorder (CUD) but stopped short of including it in substance use disorders (SUD). Instead, CUD is considered a condition needing further study, given the inconclusive research on this condition and its potential impact. Indeed, even the proposed CUD diagnosis criteria are more conservative than for other SUDs, partly because the American Psychiatric Association indicates that the extent to which this disorder is clinically significant is still unclear [187]. According to the DSM-V, at least the three following proposed criteria must be met for consideration of CUD diagnosis: 1) a persistent desire or unsuccessful effort to limit use, 2) continued use despite knowledge of potential harm, and 3) withdrawal.

In contrast to this, the International Classification of Diseases [188]recognized caffeine dependence syndrome as a condition consisting of behavioral, cognitive, and physiological changes occurring after long-term caffeine use. This nomenclature likely added to the impression that caffeine fits the criteria for addiction. However, this diagnosis has since been removed from the updated ICD-11 [189] and, thus, is more consistent with current DSM-V classifications.

While some have noted weak evidence for aspects of the DSM-V criteria (including for intoxication) and only partial support for modulation of dopamine (DA) receptors to fit the classic reward mechanism of addictive substances, they argue that the withdrawal symptoms are significant enough to warrant consideration of caffeine as a potential drug of abuse [190], with the prevalence in the general population of caffeine users estimated between 10–55% [191]. However, Heinz et al. [192] provide a solid and compelling argument for why withdrawal symptoms, along with increased tolerance, are not enough to merit the classification of caffeine as addictive. There are often counter-adaptive effects to effective pharmaceuticals, even if they are not drugs of abuse or considered addictive [193]. For example, the sudden cessation of anti-hypertensive medications, such as beta-blockers or even blood thinners, can create a critical disruption to homeostasis established during chronic use of the drug. This rapid change can have significant and very serious physiological withdrawal effects. This is also the case with certain anti-depressants [194]. Similarly, many non-addictive drugs require an increase in dosage over time due to tolerance development [192].

Another important consideration in defining an SUD and the corresponding addiction is related to the drug effects on glutamatergic and GABAergic neurotransmission as well as DA release on the ventral striatum (particularly the nucleus accumbens), resulting in drug craving, seeking, and taking despite adverse consequences [192,195]. However, it appears that caffeine does not induce DA release in the ventral striatum. It works as a competitive agonist of adenosine receptors and serves to inhibit both the A1R and A2AR. In fact, the signaling between and within the A2AR and the dopaminergic system provides a mechanism via which adenosine receptors influence behavior [196]. Increased DA D2/D3 receptor availability with caffeine administration provides further support that increases in DA are not in the striatum, as this would result in decreases in D2/D3 receptors [197].

***In summary, though it appears that caffeine has the potential to be abused by some and has established withdrawal symptoms, current mechanistic evidence and SUD criteria do not fully support the classification of caffeine as addictive. The potential for caffeine dependency or CUD may be revisited in future DSM and ICD guidelines as additional research evidence becomes available.***

## 15. Does waiting 1.5–2.0 hours after waking to consume caffeine help you avoid the afternoon “crash?”

There has been a trend, mostly on social media, to recommend delaying coffee ingestion in the morning by anywhere from 30–60 minutes to as much as 90–120 minutes after waking. The primary rationales are to prevent prolonging the waking cortisol peak because adenosine levels are still declining during this time, and/or to avoid an afternoon “crash” that some claim happens as caffeine is eliminated from the system. However, the validity and utility of these claims are questionable at best and, in some cases, not supported at all based on the available scientific evidence.

Cortisol’s circadian rhythm demonstrates a pattern where circulating levels are lowest at sleep onset and then begin to increase during sleep (typically around 0200 to 0400 h), with an eventual peak within approximately one hour of waking before declining across the day, with some stability typical in the early-afternoon hours [198]. Additional peaks also occur around meals. This is a robust effect governed by the hypothalamic-pituitary-adrenal (HPA) axis, which also helps regulate alertness and modulate sleep [199]. Interestingly, one of the hallmarks of adrenal insufficiency is early morning fatigue [200]. Caffeine does have the ability to alter the activity of the HPA axis by increasing ACTH and cortisol secretion at rest and during psychological stress [201,202], with individuals at risk for hypertension apparently particularly sensitive to this effect [202]. Lovallo et al. [203] found that 3.3 mg/kg caffeine ingestion in the morning resulted in elevated ACTH from 30–180 min post and elevated cortisol from 60–120 min post, with these levels approximately 33% and 30% higher than placebo, respectively. It has been suggested that an increased epinephrine response due to caffeine may contribute to this effect [203,204]. Of note, ingestion did not occur immediately upon waking. In other words, the increase in cortisol secretion with caffeine will still happen if you delay intake.

Of potential importance is the fact that the elevation in cortisol secretion with caffeine appears to be blunted in habitual users, even if daily intake is relatively low (~200 mg/day) [205]. In those with high chronic intakes (300–600 mg/day), this cortisol response may be abolished completely [206,207]. There is also evidence that this increase in cortisol may be limited to the morning hours as this same effect has not consistently been seen with afternoon ingestion [205], perhaps suggesting a “priming” effect of the HPA axis during periods where ACTH response may be more sensitive. It is also important to recognize that, even with HPA response to caffeine, there is no evidence that the normal diurnal pattern of cortisol is altered across the day [205]. Additionally, this maintenance of circadian rhythm with caffeine intake also appears to apply to melatonin secretion, a key regulator in the sleep-wake cycle [208]. One considerable flaw in the rationale for delaying caffeine intake based on concerns with prolonging the peak secretion of cortisol is the fact that this same response happens with high-intensity exercise when done shortly after waking [209]. Using this same logic, one would then need to suggest that this should be avoided as well, which runs contrary to almost all current evidence.

Like melatonin, adenosine also serves as a key regulator of the circadian clock and the sleep-wake cycle [210]. Adenosine itself has been speculated to serve as a homeostatic regulator of energy in the brain during sleep [211], and its formation changes in an activity-dependent manner upon waking and in certain phases (i.e. REM) of sleep due to the breakdown of ATP with increasing energetic demands [212,213]. As such, adenosine may indicate neuronal energy use, with a rapid replenishment of ATP during the initial hours of sleep as recovery and reduced metabolism dominate [214]. Upon waking, there is an immediate but gradual buildup of adenosine, while sleep causes a matching, fairly rapid exponential reduction [212]. Changes in adenosine influence wake-sleep transitions as well as sleep intensity [212]. Furthermore, the changes in adenosine in response to increased vigilance (upon waking and in response to stimuli) occur on the order of minutes, not hours [210]. There is a rapid increase in adenosine in the transition from sleep to waking, which then stabilizes across active hours [215]. The inverse response occurs at sleep onset, with a rapid reduction in the first couple of hours followed by a plateau [211,212]. In light of this pattern, any suggestion that adenosine levels are continuing to decline upon waking demonstrates a lack of understanding of the sleep-wake cycle influence on adenosine and would form a poor basis for recommending delayed caffeine intake for 90–120 minutes after waking.

Given the established circadian patterns of cortisol secretion and HPA activity, as well as adenosine accumulation and clearance, a fundamental basis for suggesting that delaying caffeine intake in the early waking hours would prevent an afternoon “crash” is completely lacking. On the contrary, there is evidence to suggest that daily “typical” caffeine intake was not associated with daytime sleepiness [211]. Even if this were to occur, a simple resolution would be an additional dose of caffeine in the early afternoon, which, as noted above, does not appear to result in negative HPA or sleep-wake cycle alterations. If anything, delaying intake would simply push the need for an afternoon dose later, which could cause sleep disruption [210].

If there is a valid reason to delay caffeine intake upon waking, it would simply have to do with effectiveness. As one purpose of sleep is to restore energy for the brain [211] with concomitant reductions in adenosine occupation of A_1_ and A_2A_ receptors, the need for a cup of coffee upon rising would be at its lowest point of the day. The exception to this would be under conditions of high sleep pressure due to such things as sleep deprivation or insufficient sleep. Sleep deprivation can alter the adenosine recovery pattern and magnitude [211], with a possible mechanism being a decline in adenosine transport resulting in increased extracellular adenosine [216]. Caffeine has also been shown to be most effective for alleviating subjective sleepiness after sleep restriction [210], which is increasingly common in modern society. As a potent adenosine antagonist, it has been demonstrated that caffeine can displace adenosine from as much as 50% of A_1_ receptors in higher doses (i.e. the equivalent of 4–5 cups of coffee) [217]. Under these conditions, a more “immediate” dosing of caffeine upon waking would likely be beneficial.

***In summary, though there may be an upside to delaying morning caffeine intake under conditions of sufficient sleep, this has to do with the magnitude of effect rather than proposed mechanisms related to prolonging the cortisol peak, continued declines in adenosine, or avoiding an afternoon “crash.” A significant drawback in the argument related to cortisol is that a similar effect occurs with intense resistance exercise performed soon after waking. Following this line of reasoning would imply that this type of early morning exercise should be avoided; however, this notion makes no scientific or pragmatic sense. The suggestion that adenosine continues to decline upon waking is also scientifically inaccurate and not supported by research. There is also no evidence that caffeine ingestion upon waking is somehow responsible for an afternoon “crash” or that delaying consumption would somehow prevent this if it did occur.***

## 16. Conclusions

1. **Does caffeine dehydrate you at rest?** Recent research has revealed that moderate daily caffeine doses (~3 mg/kg or approximately 250-300 mg) do not appear to increase urine volume in habitual caffeine consumers. On the contrary, much higher doses (6 mg/kg or more than 500 mg) may induce acute elevation of urine output. Nevertheless, the typical consumption of caffeine (i.e. usually in a beverage) has little to no effect on fluid balance.
2. **Does caffeine dehydrate you during exercise?** Factors such as sweat rate, fluid replacement, and genetic factors have a greater impact on one’s hydration level compared to moderate caffeine consumption alone. Because caffeine is a potent ergogenic aid, any issues vis a vis dehydration is likely not the result of caffeine consumption but rather fluid replacement during exercise.
3. **Does caffeine decrease body weight and fat mass?** There are a multitude of confounding variables vis a vis the effects of caffeine on weight loss. One issue is the lack of consistency in dietary intake standardization. Another variable to consider is whether the participants were caffeine-naïve before the study. Additionally, the dose of caffeine used and whether participants were overweight or not can be contributing factors. Therefore, the current body of evidence does not support the use of caffeine as a fat loss aid.
4. **Does habitual caffeine consumption influence the performance response to acute caffeine supplementation?** The majority of available evidence indicates that regular caffeine consumption does not have an adverse influence on performance after a single dose of caffeine is administered. Data suggests that a dosage of 6-9 mg/kg body mass may be needed to produce a performance-enhancing effect. Nevertheless, because most studies have not reported on participants’ regular caffeine intake, further research is necessary to draw more definitive conclusions.
5. **Does caffeine affect upper vs. lower body performance/strength differently?** The performance-enhancing effects of caffeine on upper versus lower body strength are contingent on factors like dosage, individual variances, muscle group size, and the type of activity. The preponderance of evidence suggests that acute caffeine consumption does not affect the lower vs. upper body differently.
6. **Is there a relationship between caffeine and depression?** While moderate caffeine intake can offer temporary relief from certain depressive symptoms and even potentially improve mood for some individuals, excessive consumption of caffeine can worsen anxiety, disturb sleep, and result in adverse mental health consequences. It is essential for those dealing with depression to be aware of how caffeine affects their overall well-being and to seek professional guidance when needed.
7. **Can too much caffeine kill you?** Although quite rare, caffeine can be fatal in cases of overdose; such circumstances are generally not applicable to healthy individuals who typically consume caffeine via beverages such as tea or coffee.
8. **Are there sex differences regarding caffeine’s effects?** The literature surrounding sex differences in caffeine metabolism and subjective effects of caffeine are mixed. Nonetheless, caffeine is an effective ergogenic aid, and even the placebo effect of caffeine improves sports performance irrespective of sex, with some studies reporting greater performance advantages in males.
9. **Does caffeine work for everyone?** It is evident that there is substantial variability among individuals in their response to caffeine’s performance-enhancing effects. While it is possible that some people may not respond to caffeine, such non-responders appear to be infrequent. Further investigations involving multiple assessments with various tasks and dosing approaches are needed to definitively establish an individual’s responsiveness to caffeine.
10. **Does caffeine cause heart problems?** An individual’s overall cardiovascular risk profile in relation to caffeine is likely to be shaped by a variety of factors. These factors include the amount consumed, how long it has been consumed, the way it is consumed (e.g. through coffee or tea), and an individual’s metabolic and genetic variations. It is worth noting that a significant portion of caffeine intake comes from coffee, which is associated with various health benefits. There is no evidence that low to moderate intake of caffeine has adverse effects on cardiac muscle.
11. **Does caffeine promote the loss of bone mineral?** The evidence is equivocal. There is evidence to suggest that ≤ 4 cups of coffee (≤400 mg of caffeine) is the threshold for concern regarding BMD and/or fracture risk, primarily in females. However, additional clinical research is needed to explore the potential dose-response relationship between caffeine consumption and bone health and investigate relevant confounding variables (i.e. diet, population, and the delivery form of caffeine [e.g. coffee, tea, soft drinks, energy drinks, or caffeine alone]) before drawing any firm conclusions.
12. **Should pregnant women avoid caffeine?** A review of the available literature shows that the substantial majority of findings from observational studies and meta-analyses is that maternal caffeine consumption is reliably associated with major negative pregnancy outcomes.
13. **Is caffeine addictive?** Although it appears that caffeine has the potential to be abused by some and has established withdrawal symptoms, current mechanistic evidence and SUD criteria do not fully support the classification of caffeine as addictive.
14. **Does waiting 1.5-2.0 hours after waking to consume caffeine help you avoid the afternoon “crash?”** There is no evidence that caffeine ingestion upon waking is somehow responsible for an afternoon “crash” or that delaying consumption would somehow prevent this if it did occur.
